# Analysis of Strain Field Heterogeneity at the Microstructure Level and Inverse Identification of Composite Constituents by Means of Digital Image Correlation

**DOI:** 10.3390/ma13020287

**Published:** 2020-01-08

**Authors:** Witold Ogierman, Grzegorz Kokot

**Affiliations:** Department of Computational Mechanics and Engineering, Faculty of Mechanical Engineering, Silesian University of Technology, Konarskiego 18a Street, 44-100 Gliwice, Poland; grzegorz.kokot@polsl.pl

**Keywords:** Digital Image Correlation (DIC), composite materials, micromechanics, inverse problem, finite element method (FEM), mechanical properties

## Abstract

The present paper is devoted to the theoretical study on the estimation of the full-field strain at the microstructural level of composite materials by means of Digital Image Correlation (DIC). The main aim of the paper is to investigate the influence of speckle size on the accuracy of the strain field measurement at the microscale. The DIC analysis was conducted based on artificial speckle patterns generated numerically and the deformation behavior of the composites was simulated by using the finite element method (FEM). This approach gives the opportunity to compare the results of the DIC in terms of speckle size with the reference FEM solution. Moreover, the paper focuses on the inverse identification of the material constants of the composite constituents by using information associated with the measured strain field. The inverse problem is solved by using a novel two-step optimization procedure, which reduces the problem complexity. The feasibility and accuracy of the proposed approach are presented by analysis of two exemplary microgeometries representing the microstructures of fiber reinforced composites.

## 1. Introduction

Effective mechanical properties of composite materials depend on the material properties of the constituents, the shape and volume fraction of the reinforcement as well as the interactions between the reinforcement and the matrix. The strain field at the microscopic level is inhomogeneous due to the contrast between the matrix and reinforcement material properties. The strain field heterogeneity can be analyzed by using various analytical, numerical and experimental methods. Analytical micromechanical models based on Eshelby’s solution [1] such as Mori–Tanaka [2,3] or self-consistent [4,5] methods allow the estimation of the strain partitioning between the matrix and reinforcement by referring to the per phase average strains. On the other hand, when analytical methods are used, it is difficult to consider strain concentrations which are effects of fibers clustering and nonuniform spatial distribution. The other way is to use numerical methods which can take into consideration complex geometrical models describing the material microstructure. The most versatile and widely used numerical method for analysis of the deformation behavior of composites at the microstructural level is the finite element method (FEM) [6,7,8,9]. The other numerical methods which can be used for this purpose are the boundary element method (BEM) [10,11] and meshless methods [12,13]. Recently also experimental optical methods have been applied for measuring the deformation behavior at the microstructural level. In this case the most popular method is digital image correlation (DIC) which is a computer-based procedure that allows full-field displacement information to be obtained by recording the motion of a speckle pattern on a specimen surface during the deformation [14,15,16,17]. The numerical procedures of the DIC are based on pseudo-affine transformation where series of pictures collected during the deformation of the specimen are correlated and results are presented in the form of full field color maps of the displacements or strains [18]. The DIC can be applied for different length scales since the method has no inherent length scale [14]. However, the accuracy of the DIC method depends on the quality of the speckle pattern which must be placed on the specimen surface [19,20]. Literature discusses several different approaches to the preparation of the speckle pattern for DIC testing at the microscale. The feasibility of the DIC technique for the measurement on micro- and nanoscale was demonstrated by Berfield et al. [14], the random speckle pattern was created by a fine point airbrush (for microscale measurement) and by solution deposition of fluorescent silica nanoparticles (for nanoscale measurement). Ghadbeigi et al. measured the strain field at the microstructure level of dual-phase steel [21] and interstitial-free steel [22], the authors used the natural pattern of the microstructure in order to carry out the DIC analysis. Anzelotti et al. [23] studied the behavior of a twill-weave carbon fiber reinforced epoxy lamina, the surface was prepared for the DIC measurement by using a white ink spray. Canal et al. [24] and Mehdikhani et al. [25] analyzed the behavior of fiber-reinforced composites, the speckle patterns were created by depositing submicron alumina particles. Joo et al. [26] measured local strain in a dual phase steel by DIC by basing on the nanodot patterns.

The present paper is devoted to theoretical study on the influence of the speckle size on the accuracy of the strain field measurement at the microstructural scale by means of DIC. This is a very important issue from the practical point view because before capturing the images of undeformed and deformed specimen a speckle pattern that guarantees exposing the strain field heterogeneity due to the underlying microstructure is required. Theoretical estimation of the required speckle size can provide information on how the speckle pattern should be prepared, or which preparation method should be used. On the other hand, the error of measurement caused by the non-optimal speckle pattern can be quantified. The paper presents a numerical procedure of the generation of artificial speckle patterns and the technique of mapping of these patterns on a virtually deformed specimen. This work concentrates on unidirectional long fiber reinforced composites. The accuracy of DIC analysis with respect to different sizes of the speckles is investigated by analyzing microgeometries containing several fibers. Moreover, the paper presents a novel approach of inverse identification of the properties of the composite constituents. The idea is to perform an inverse identification of the elastic constants of the composite constituents by basing on the full strain field measurement. This way of indirect estimation of material properties may be very useful for quantification of the properties of individual phases of composites which are difficult to capture during standard experimental testing, for instance in the case of in situ composites. There are several works which are devoted to usage of full field measurement for the purpose of inverse identification of elastic properties of materials at the macroscopic level (constituents are not distinguished in this case) [27,28,29,30,31]. One of the most widely used identification approaches is the finite element model updating method which is based on minimizing the discrepancy between the displacements measured experimentally and the displacements computed by the FEM depending on the material constants (variables). Rahmani et al. [32] used the full field measurement data for identification of the elastic constants of composite constituents, the authors improved the finite element model updating method by adding a regularization term which assumes that besides the displacements, the results provided by the micromechanical model are also fitted to the experimental data. The present paper introduces a novel approach of inverse identification which is based on minimizing the discrepancy between the per phase average strains measured experimentally and computed by using FEM. The inverse problem is solved in a framework of new two-step optimization procedure, which reduces the problem complexity. The feasibility and accuracy of the proposed approach are presented by analysis of two exemplary microgeometries representing the microstructures of fiber reinforced composites.

## 2. Materials and Methods

### 2.1. Artificial Speckle Patterns

**Figure 1:**
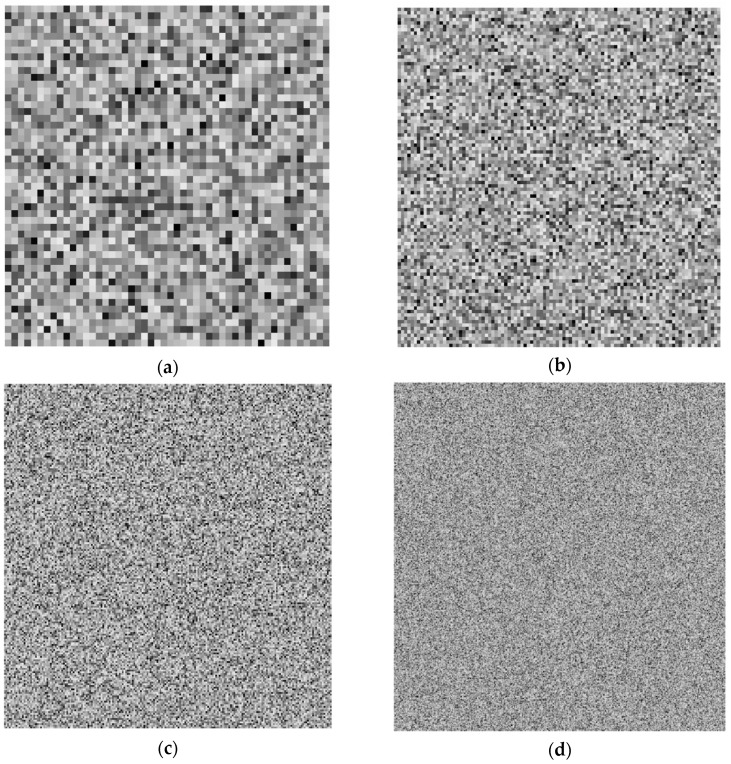
Artificial speckle patterns generated with respect to different speckle sizes, grids of squares: (**a**) 50 × 50 (**b**) 100 × 100; (**c**) 200 × 200; (**d**) 400 × 400.

**Figure 2:**
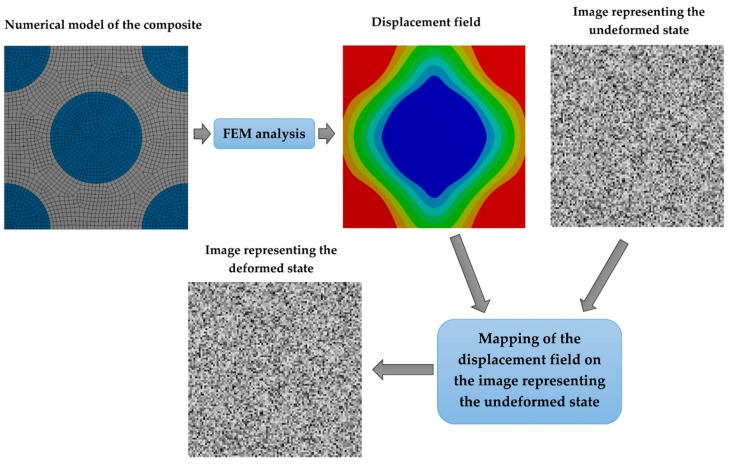
Scheme of generation of artificial speckle patters representing the deformed state.

In order to investigate the influence of the speckle size on the accuracy of the DIC analysis artificial speckle patterns were generated. The procedure of the pattern generation assumes that the image consists of a predefined number of squares (grid of squares) whose grayscale intensity is determined in a random manner. Exemplary speckle patterns associated with different sizes of the speckles are presented in Figure 1. A more challenging issue is the preparation of artificial speckle patterns which relate to the specimen after deformation, Figure 2 presents a scheme of generation of such images. The first step is associated with creation of the FE model which describes the geometry and constitutive behavior of the material phases as well as the boundary conditions. The result of the FE analysis is a displacement field. Afterwards the displacement field is interpolated to the corners of the squares that built the artificial speckle patterns representing the undeformed state. The coordinates describing a location of the corners of the squares are respectively modified, in consequence the deformed state is represented by an image consisting of a set of quadrilaterals. Finally, the images representing the undeformed and deformed state can be treated as input data for the DIC analysis.

### 2.2. Inverse Identification Problem

The aim of the identification procedure is determination of the per phase material properties by basing on the data captured during experimental testing of the composite material. In the case of a two phase composite the variables are the Young’s moduli of matrix E_m_ and fiber E_f_ and the Poisson ratios of matrix υ_m_ and fiber υ_f_ respectively. The method proposed in this paper assumes that identification is carried out by solving the optimization problem associated with minimization of the discrepancy between the per-phase average strains measured experimentally and computed by using the theoretical model. The objective function can be defined in the following way:

(1) $$\min\;F(E_{m},E_{f},\upsilon_{m},\upsilon_{f})=\sum_{i=1}^{2}\sum_{j=1}^{2}\left[{\left({\langle \varepsilon_{\mathrm{ij}}\rangle }_{m}^{\mathrm{THE}}-{\langle \varepsilon_{\mathrm{ij}}\rangle }_{m}^{\mathrm{DIC}}\right)}^{2}+{\left({\langle \varepsilon_{\mathrm{ij}}\rangle }_{f}^{\mathrm{THE}}-{\langle \varepsilon_{\mathrm{ij}}\rangle }_{f}^{\mathrm{DIC}}\right)}^{2}\right],$$

where superscript DIC denotes the experimental results and superscript THE denotes the results obtained by using the theoretical model. The described optimization problem can be simplified by dividing the optimization procedure into two steps. In the case of two phase composites the strain field depends on the υ_m_, υ_f_ and relationship between the Young’s moduli:

(2) $$R=\frac{E_{f}}{E_{m}},$$

therefore, the number of variables can be reduced to three. The optimization constraints associated with the lower and upper bounds for the variables provided in the following way:

(3) $$R\in \;\langle 1,500\rangle {\;\mathrm{and}\;\upsilon }_{f},\upsilon_{m}\in \;\langle 0.1,0.45\rangle .$$

should be suitable for most of composite materials. The described problem is ill-posed in the Hadamard sense, therefore in order to find a unique solution an appropriate regularization term must be included. The DIC measurement of strains at the microscale is performed in the plane xy however the additional physical information may be associated with the effective (composite) out-of-plane properties. For example, Rahmani et al. [32] used out-of-plane stiffness as the additional information. In order to extend the objective function described by Equation (1) it is more convenient to add information related to the transverse Poisson ratio υ_c_. It is worth mentioning that it could be determined straightforwardly for instance by using a standard tensile test including two extensometers [33] or DIC (at the macroscopic level of composite) [33,34]. The discrepancy between the transverse Poisson ratio for the composite determined experimentally and theoretically can be expressed in the form of an additional term included in the objective function. In consequence the objective function for the first optimization step can be formulated as follows:

(4) $$\min\;F(R,\upsilon_{m},\upsilon_{f})=\sum_{i=1}^{2}\sum_{j=1}^{2}\left[{\left({\langle \varepsilon_{\mathrm{ij}}\rangle }_{m}^{\mathrm{THE}}-{\langle \varepsilon_{\mathrm{ij}}\rangle }_{m}^{\mathrm{DIC}}\right)}^{2}+{\left({\langle \varepsilon_{\mathrm{ij}}\rangle }_{f}^{\mathrm{THE}}-{\langle \varepsilon_{\mathrm{ij}}\rangle }_{f}^{\mathrm{DIC}}\right)}^{2}\right]+{\left(\upsilon_{c}^{\mathrm{THE}}-\upsilon_{c}^{\mathrm{EXP}}\right)}^{2}.$$

**Figure 3:**
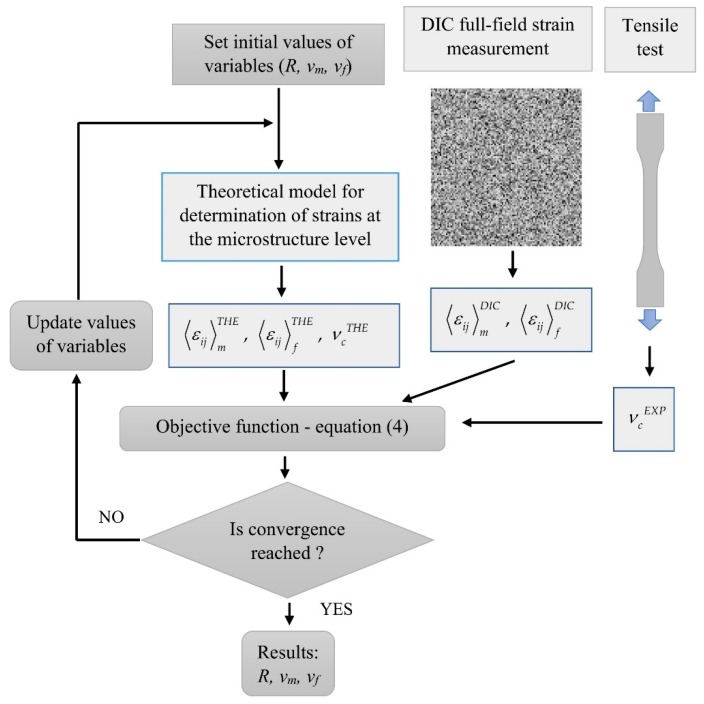
Scheme of the first optimization step.

The first optimization step is illustrated schematically in Figure 3.

To solve the described optimization problem a global optimization method like for example a genetic algorithm can be applied [35,36,37,38,39]. In the case of the theoretical model, naturally the FE model which reflects the actual microstructure can be used. Here, the strains associated with the matrix and inclusion can be obtained by averaging over the volume of the domain occupied by the matrix dΩ_m_:

(5) $${\langle \varepsilon_{\mathrm{ij}}\rangle }_{m}^{\mathrm{FEM}}=\int_{\Omega_{m}}\varepsilon_{\mathrm{ij}}(E_{m},E_{f},\upsilon_{m},\upsilon_{f})d\Omega_{m},$$

and the volume of the domain occupied by the fiber dΩ_f_:

(6) $${\langle \varepsilon_{\mathrm{ij}}\rangle }_{f}^{\mathrm{FEM}}=\int_{\Omega_{f}}\varepsilon_{\mathrm{ij}}(E_{m},E_{f},\upsilon_{m},\upsilon_{f})d\Omega_{f}.$$

The FE based modeling of the deformation behavior of the microstructure can provide very good accuracy however it should be noted that it may lead to very long times of computations due to many objective function evaluations which are required by the global optimization algorithm. Moreover a two-dimensional FE model used during the optimization is not able to provide the transverse Poisson ratio thus a three-dimensional FE model should be applied which in turn may lead to prohibitive times of computations. On the other hand, the proposed objective function formulated in terms of per-phase average fields allows the application of an efficient mean field homogenization model instead of FEM. During this work the Mori–Tanaka model [2,3,40,41,42,43,44], which found wide popularity due to good predictive capabilities, was used. In this case the strain average over the composite is related to the strain averages over the matrix and fiber in the following way:

(7) $${\langle \varepsilon \rangle }_{c}={\langle \varepsilon \rangle }_{m}\left(1-f_{f}\right)+{\langle \varepsilon \rangle }_{f}f_{f},$$

where f_f_ is a volume fraction of the fibers. A relationship between the strain average over the matrix and fibers can be expressed in terms of the strain concentration tensor A:

(8) $${\langle \varepsilon \rangle }_{f}={\left[S\left(C_{m}{}^{-1}C_{f}-I\right)+I\right]}^{-1}{\langle \varepsilon \rangle }_{m}\equiv A{\langle \varepsilon \rangle }_{m}$$

where S is the Eshelby tensor, C_m_ and C_f_ are elastic stiffness tensors for matrix and fiber respectively. The Eshelby tensor depends on the Poisson ratio of the matrix and shape of the inclusion, components of these tensors for continuous fiber can be found for example in the book of Mura [45]. The strain averages over the fiber and the matrix depend on the strain average over the composite in the following way:

(9) $${\langle \varepsilon \rangle }_{f}=A{\left[f_{f}A+\left(1-f_{f}\right)I\right]}^{-1}{\langle \varepsilon \rangle }_{c},\;{\langle \varepsilon \rangle }_{m}={\left[f_{f}A+\left(1-f_{f}\right)I\right]}^{-1}{\langle \varepsilon \rangle }_{c}$$

The effective stiffness tensor for the composite can be expressed in the following form:

(10) $$C=C_{m}+f_{f}(C_{f}-C_{m})A{\left[(1-f_{f})I+\mathrm{fA}\right]}^{-1}$$

After identifying the υ_m_, υ_i_, and R the second optimization step, whose aim is to determine the E_m_ and E_i_ must be carried out. There is only one variable during the second optimization step since R is known. The objective function is determined as the minimization of the stress average over the composite domain computed by using the theoretical model and stress determined experimentally:

(11) $$\min\;F(E_{m})={\left({\langle \sigma_{11}(E_{m})\rangle }_{c}^{\mathrm{THE}}-{\langle \sigma_{11}\rangle }_{c}^{\mathrm{EXP}}\right)}^{2}$$

It is worth mentioning that the experimental determination of stress (in plane xy) can be carried out by using the force transducer integrated with the loading device (during the microscopic observation). The other way, which also provides accurate results, is comparison of the longitudinal Young’s modulus E_L_ determined experimentally and theoretically (experimental data can be captured during the transverse Poisson ratio measurement carried out for the purpose of regularization):

(12) $$\min\;F(E_{m})={\left(E_{L}{\left(E_{m}\right)}^{\mathrm{THE}}-E_{{}_{L}}^{\mathrm{EXP}}\right)}^{2}.$$

**Figure 4:**
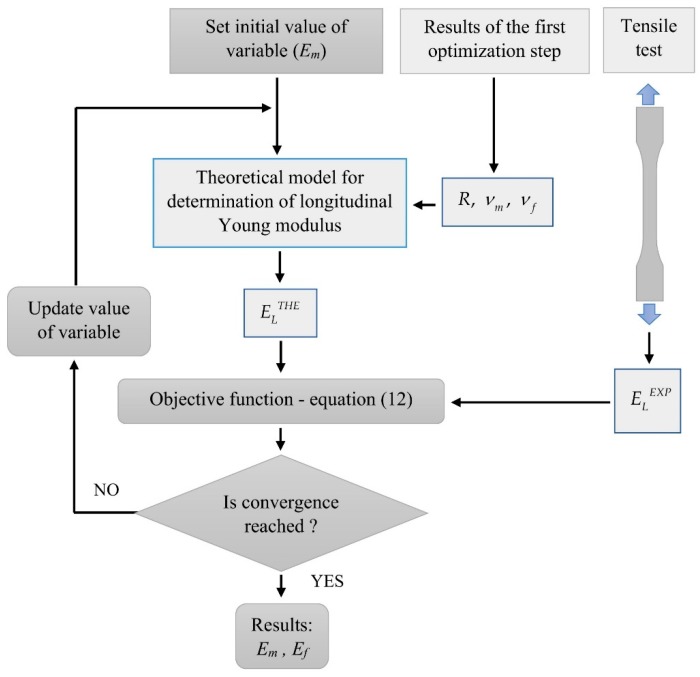
Scheme of the second optimization step.

The problem related to the second step is well-posed and can be solved by using a gradient based optimization method very efficiently. The second optimization step is illustrated schematically in Figure 4.

## 3. Results and Discussion

### 3.1. Numerical Modeling of the Deformation Behavior

**Figure 5:**
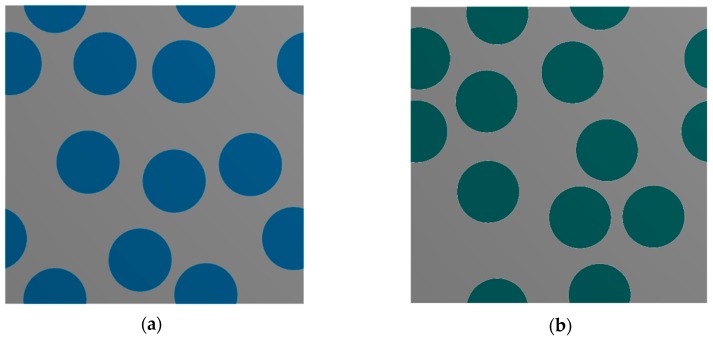
Geometrical models of the composite microstructure: (**a**) Material 1; (**b**) Material 2.

**Figure 6:**
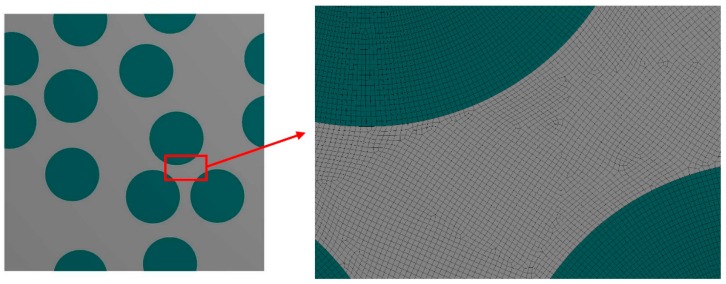
Finite element mesh discretizing the microgeometry.

**Figure 7:**
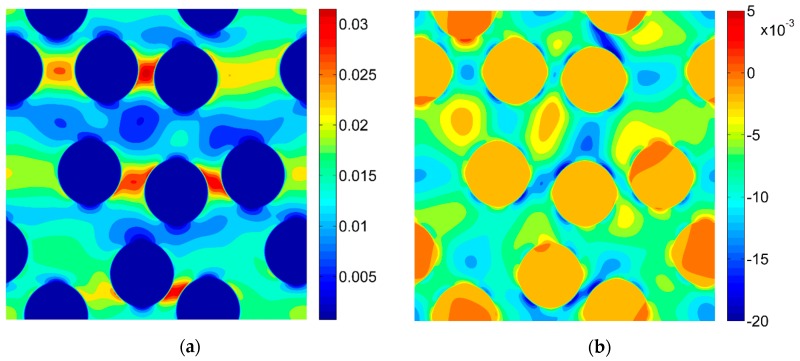

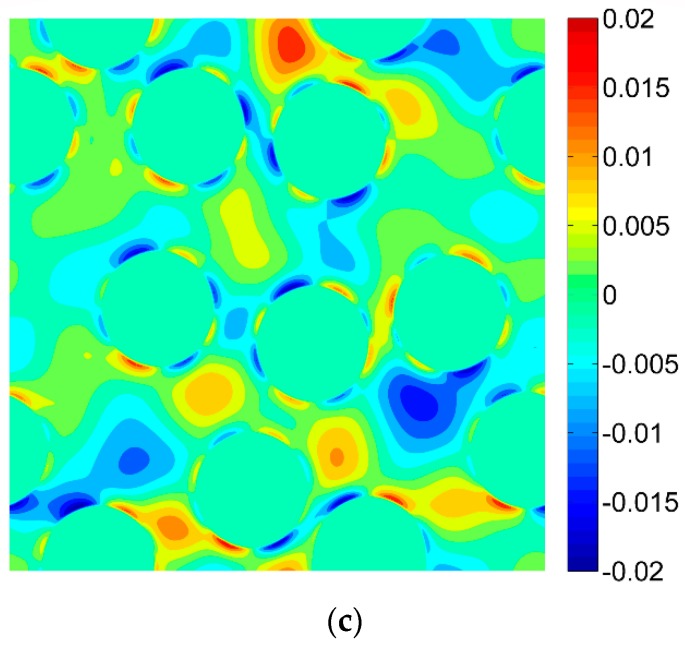
Strain fields obtained by using the finite element method (FEM) for Material 1 (**a**) ε_11_; (**b**) ε_22_; (**c**) ε_12_.

**Figure 8:**
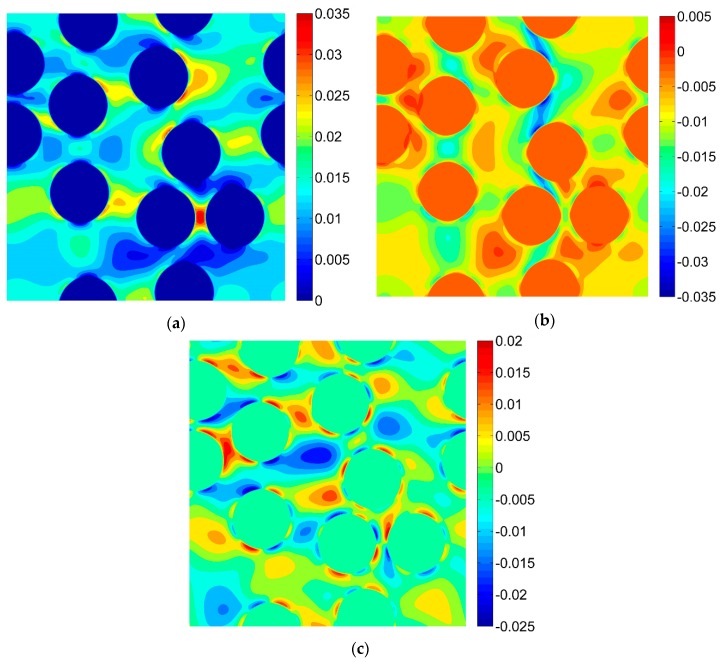
Strain fields obtained by using the FEM for Material 2 (**a**) ε_11_; (**b**) ε_22_; (**c**) ε_12_.

For the purpose of the virtual tests, two different microgeometries representing the microstructures of the composites were taken into account (Figure 5). Two-dimensional, plane strain finite element models were considered. In order to perform the FE computations ANSYS software (version 18.2, Canonsburg, PA, USA) was used. The DIC can be applied for different length scales thus, no particular unit of length associated with the models of microgeometry was introduced, a length of the side of the square related to the geometrical models was considered as unitary. A fine finite element mesh consisting of quadrilateral elements was generated, the global length of the edge of the element equals 0.002 (Figure 6). Material properties describing the behavior of the constituents corresponding to the analyzed microgeometries are collected in Table 1, the volume fraction of the fibers equals 0.35. Periodic boundary conditions [46,47] enforcing uniaxial strain ε_11_ = 0.01 were applied for both microgeometries. The obtained strain fields are presented in Figure 7 (for material 1) and in Figure 8 (for material 2).

**Figure 9:**
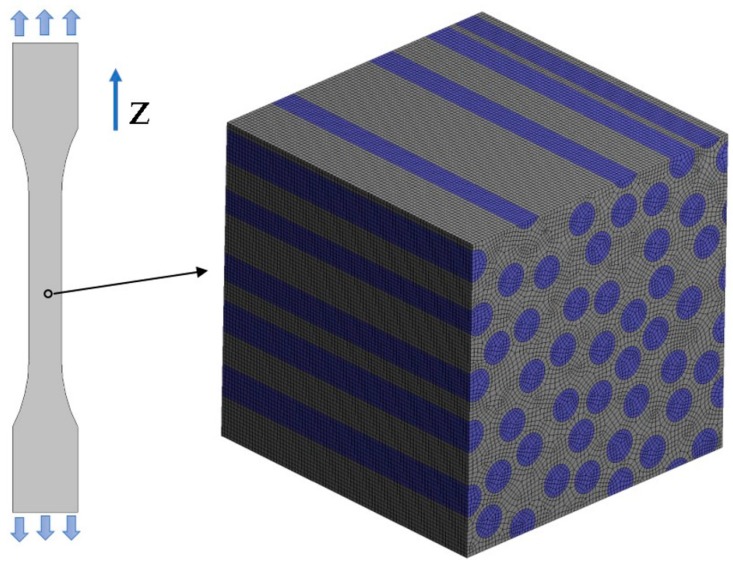
Virtual tensile test along the Z axis, representative volume element (RVE) representing the microstructure of the composite.

The measurement of the transverse Poisson ratio (required as the regularization factor) was also simulated by using the FEM. In this case, a tensile test of the composite was modeled by assuming a three-dimensional representative volume element (RVE) whose geometry is presented in Figure 9. The unit uniaxial strain ε_33_ = 1 was enforced by prescribing mixed boundary conditions, after solving the boundary value problem the effective transverse Poisson ratio can be determined in the following way:

(13) $$\upsilon_{c}=-\int_{\Omega_{c}}\varepsilon_{11}(E_{m},E_{f},\upsilon_{m},\upsilon_{f})d\Omega_{c}.$$

During the tensile test also the longitudinal Young’s modulus can be determined:

(14) $$E_{c}=\int_{\Omega_{c}}\sigma_{33}(E_{m},E_{f},\upsilon_{m},\upsilon_{f})d\Omega_{c}.$$

The Young’s moduli and Poisson ratios obtained after the FE simulations for both analyzed materials are collected in Table 2.

### 3.2. Results of the Digital Image Correlation

**Figure 10:**
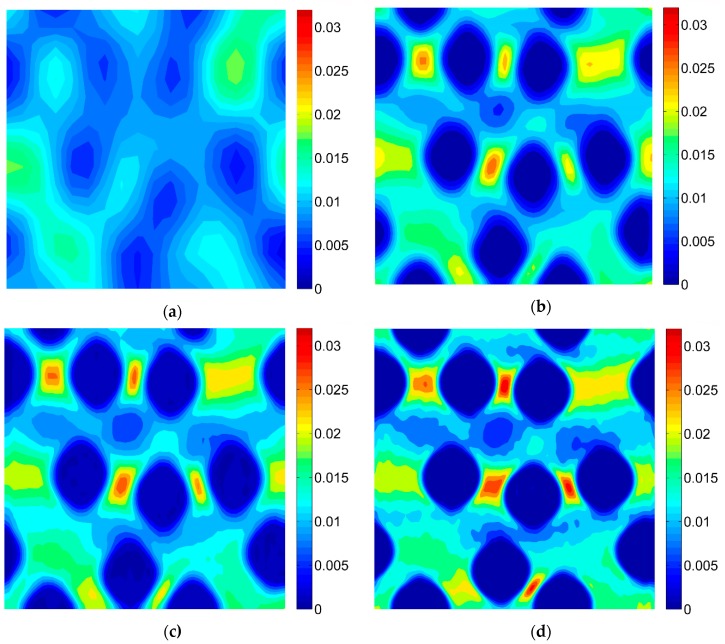
Distribution of strain ε_11_ obtained for Material 1 with consideration of different speckle sizes, grid of squares: (**a**) 50 × 50, (**b**) 100 × 100, (**c**) 150 × 150, (**d**) 200 × 200.

**Figure 11:**
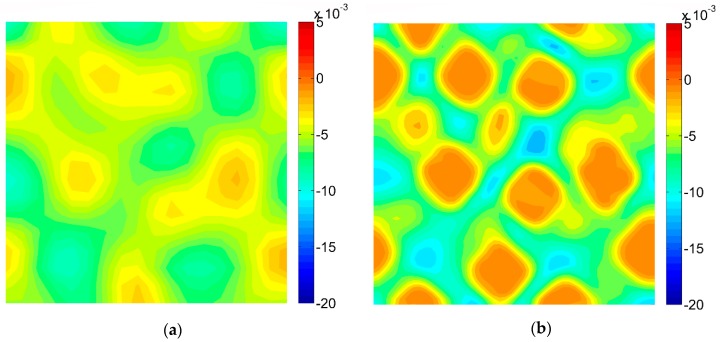

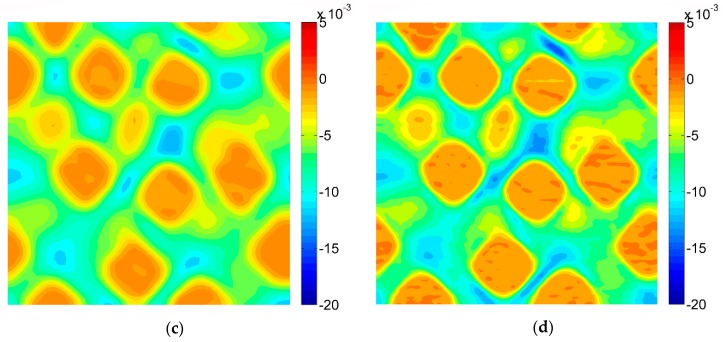
Distribution of strain ε_22_ obtained for Material 1 with consideration of different speckle sizes, grid of squares: (**a**) 50 × 50, (**b**) 100 × 100, (**c**) 150 × 150, (**d**) 200 × 200.

**Figure 12:**
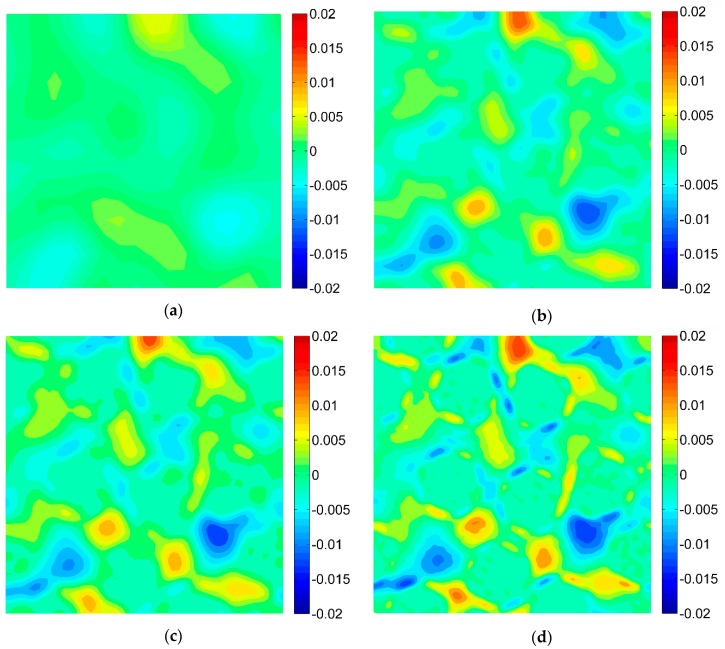
Distribution of strain ε_12_ obtained for Material 1 with consideration of different speckle sizes, grid of squares: (**a**) 50 × 50, (**b**) 100 × 100, (**c**) 150 × 150, (**d**) 200 × 200.

**Figure 13:**
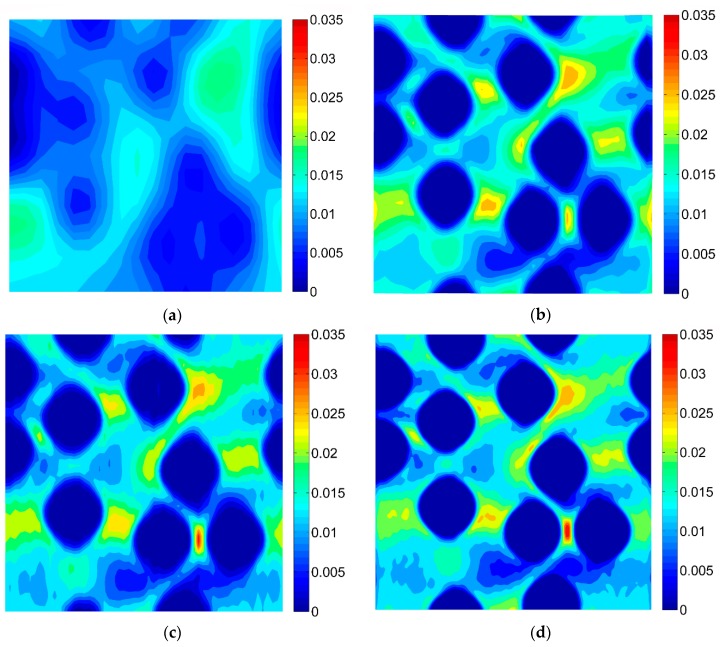
Distribution of strain ε_11_ obtained for Material 2 with consideration of different speckle sizes, grid of squares: (**a**) 50 × 50, (**b**) 100 × 100, (**c**) 150 × 150, (**d**) 200 × 200.

**Figure 14:**
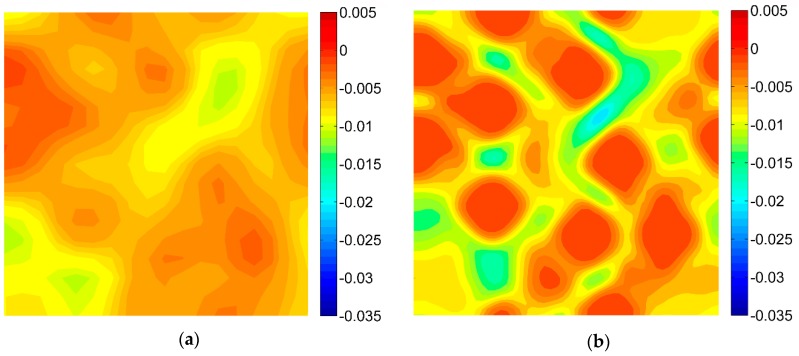

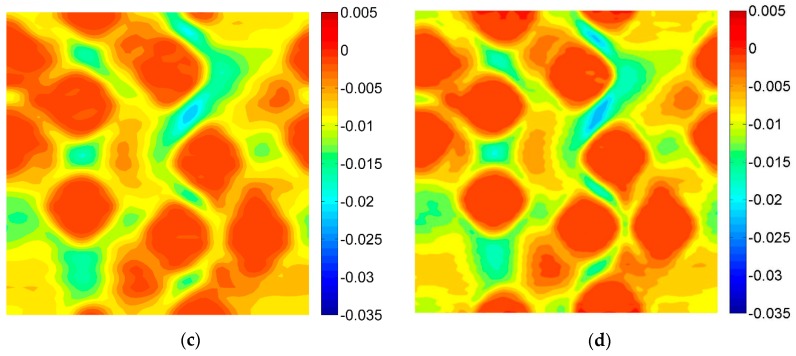
Distribution of strain ε_22_ obtained for Material 2 with consideration of different speckle sizes, grid of squares: (**a**) 50 × 50, (**b**) 100 × 100, (**c**) 150 × 150, (**d**) 200 × 200.

**Figure 15:**
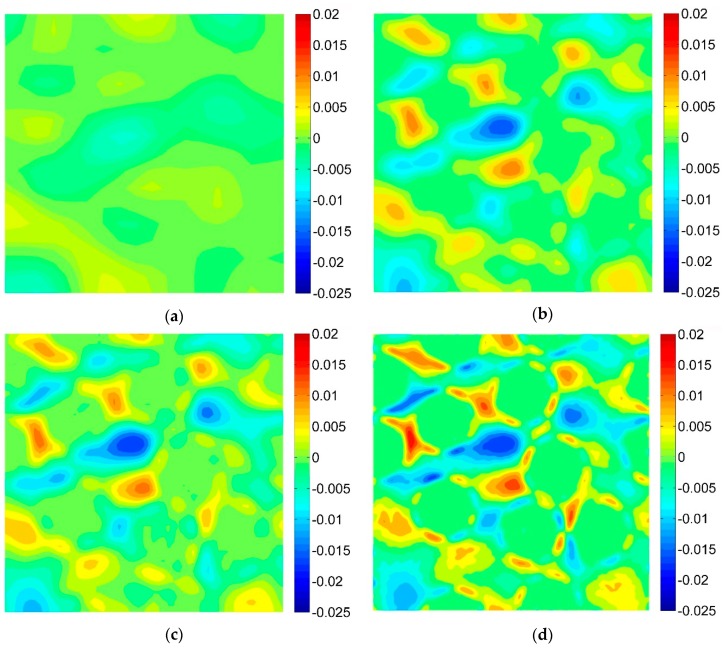
Distribution of strain ε_12_ obtained for Material 2 with consideration of different speckle sizes, grid of squares: (**a**) 50 × 50, (**b**) 100 × 100, (**c**) 150 × 150, (**d**) 200 × 200.

The DIC analysis was carried out by using ISTRA 4D software (Dantec Dynamics GmbH, Ulm, Germany). Four different speckle patterns were considered in accordance with those presented in Figure 1. The coarsest speckle pattern consisting of 50 × 50 quadrilaterals provides a ratio of the fiber diameter to the single speckle size equaling approximately 10, while the finest speckle pattern consisting of 400 × 400 quadrilaterals provides a ratio eight times higher. The results of the analysis are presented in the form of color maps illustrating the strain distributions. Figure 10, Figure 11 and Figure 12 present the distributions of ε_11_, ε_22_ and ε_12_ strains for Material 1. Figure 13, Figure 14 and Figure 15 present the distributions of ε_11_, ε_22_ and ε_12_ strains for Material 2, respectively. Moreover, the results are summarized in Table 3 and Table 4 where the average strains for matrix <ε_11_>_m_, <ε_22_>_m_ and fibers <ε_11_>_f_, <ε_22_>_f_ as well as errors related to the reference FE solution are collected.

Similar conclusions can be drawn for both analyzed materials. The coarsest speckle pattern does not allow the strain distribution to be captured properly although it indicates that the strain distribution is inhomogeneous, the positions of the fibers are exposed. Decreasing the speckle size twice (grid 100 × 100) leads to dramatically better results, in this case the strain distribution is quite similar to the reference one (FE solution) however the peak strains are significantly underestimated and the results near the fiber-matrix interface are highly averaged. Application of the finest speckle pattern provides a strain field in good agreement with the reference one, peak strains are only slightly underestimated.

### 3.3. Results of Inverse Identification

The inverse problem described in Section 2.2 was solved by using the proposed two-step optimization procedure. The solution of the first optimization step was carried out by using the genetic algorithm with consideration of a population consisting of 100 chromosomes and 30 generations which in turn led to 3000 evaluations of the objective function. The second optimization step was solved by using the gradient based algorithm, in this case 10 evaluations of the objective function formulated by Equation (12) were enough to find the optimum. The Mori–Tanaka method was used as theoretical model during evaluation of the objective function. At first, in order to verify the proposed identification procedure, the input data for identification was selected from the results obtained by using the FEM. Afterwards, the input data was taken from the results obtained by the DIC with consideration of different sizes of the speckles. The identified elastic constants are collected in Table 5 and Table 6 for Material 1 and Material 2 respectively.

An interesting finding is that the Young’s modulus of the fiber was identified with relatively high accuracy for all considered speckle sizes, the accuracy decreases only slightly with increasing size of the speckles. On the other hand, in order to obtain reasonable accuracy of identification of Young’s modulus of the matrix a fine speckle pattern must be applied, the accuracy decreases significantly with increasing size of the speckles. The Poisson ratios of matrix and fiber were identified with reasonable accuracy, in this case accuracy of the identification decreases with increasing size of the speckles more significantly than in case of the fiber Young’s modulus. However, the accuracy is much less prone to changes than in the case of the Young’s modulus of the matrix. It is worth mentioning that in some cases the results of identification obtained on the basis of data related to the DIC analysis are surprisingly more accurate than in the case of the input data related to the FEM (“exact” input data). This fact is caused by differences between the output of the Mori–Tanaka model and the FE analysis based on the microstructure geometry. In other words, discrepancy between the strain averages obtained by using the Mori–Tanaka model and the FE analysis may be larger than the discrepancy between the strain averages obtained by using the Mori–Tanaka model and the DIC analysis (which uses the FE results as input). Nonetheless, the proposed method of identification provides reasonable accuracy and a time-efficient solution.

## 4. Concluding Remarks

This paper presents a study on the influence of the speckle size on the accuracy of the strain field measurement at the microscale by means of DIC. Generation and analysis of artificial images representing the undeformed and deformed material states gave an opportunity to compare the results of the DIC in terms of the speckle size with the reference FE solution. Such analysis led to the establishment of an approximate size of the speckles which is required to observe the heterogenous strain field at the microstructure level with the desired accuracy. On the other hand, it could be a useful tool for estimation of the error level of the DIC analysis when a nonoptimal speckle pattern is used. Moreover, a novel method of inverse identification of the elastic properties of the composite constituents was proposed. Identification was performed by solving the optimization problem associated with minimization of the discrepancy between the per phase average strains measured experimentally and computed by using the theoretical model. The inverse problem was solved by using a novel two-step optimization procedure, which reduces the problem complexity. It is worth mentioning that the proposed formulation of the objective function allows the use of a time-efficient Mori–Tanaka model as theoretical model for computing the per-phase average strains which in turn leads to a very efficient solution of the inverse problem. The measured strain field at the microstructure level does not provide enough information for identification, therefore an additional regularization term, like for example the transverse Poisson ratio, must be added. The accuracy of the identification was analyzed in terms of the size of the speckles; in general the proposed method can provide reasonable accuracy of identification.

Further work will be concerned with the experimental testing of the deformation behavior of composites at the microstructural level. The method of assessment of the required speckle pattern size proposed in the present paper should be applied in order to perform reliable measurements. In this case a digital representation of a real speckle pattern covering a specimen’s surface can be considered instead of the artificial speckle pattern. Then the displacements obtained from the FE analysis of the material microstructure can be mapped on the digital representation of the real speckle pattern. Finally, the discrepancy between the reference FE solution and DIC analysis based on the digital representation of the real speckle pattern may be a useful indicator of the accuracy of the DIC analysis carried out on the basis of images captured during the experiment.

## Figures and Tables

**Table 1:** Material Properties of the Composite Constituents.

	E_m_, MPa	E_i_, MPa	υ_m_	υ_i_
Material 1	3500	72,000	0.35	0.20
Material 2	2600	115,000	0.39	0.28

**Table 2:** Elastic constants determined during the virtual tensile test.

	υ_c_	E_c_, MPa
Material 1	0.2881	27,734.2
Material 2	0.3458	42,335.2

**Table 3:** Summary of results of the DIC (Digital Image Correlation) analysis involving different speckle patterns obtained for Material 1.

	<ε_11_>_m_	<ε_11_>_f_	<ε_22_>_m_	<ε_22_>_f_
FEM (Finite Element Method), reference Solution	0.01471	0.00124	−0.00727	−0.00040
DIC, grid 400 × 400	0.01419	0.00121	−0.00697	−0.00046
-	3.7%	6.0%	4.3%	16.1%
DIC, grid 200 × 200	0.01393	0.00133	−0.00682	−0.00056
-	5.3%	6.9%	6.3%	41.9%
DIC, grid 100 × 100	0.01348	0.00139	−0.00654	−0.00052
-	8.4%	12.1%	10.0%	30.7%
DIC, grid 50 × 50	0.01169	0.00319	−0.00577	−0.00174
-	20.6%	156.3%	20.6%	340.7%

**Table 4:** Summary of results of the DIC Analysis involving different speckle patterns obtained for Material 2.

	<ε_11_>_m_	<ε_11_>_f_	<ε_22_>_m_	<ε_22_>_f_
FEM, reference solution	0.01505	0.00063	−0.00839	−0.00033
DIC, grid 400 × 400	0.01442	0.00061	−0.00796	−0.00031
-	4.2%	2.5%	5.1%	5.9%
DIC, grid 200 × 200	0.01377	0.00060	−0.00762	−0.00037
-	8.5%	4.1%	9.2%	13.7%
DIC, grid 100 × 100	0.01343	0.00059	−0.00740	−0.00037
-	10.8%	5.8%	11.7%	13.6%
DIC, grid 50 × 50	0.01155	0.00178	−0.00650	−0.00180
-	23.2%	183.2%	22.5%	454.4%

**Table 5:** Results of the Identification of Constituents Properties for Material 1.

	υ_m_	υ_f_	R	E_m_, MPa	E_f_, MPa
FEM-reference solution	0.3500	0.2000	20.6	3500.0	72,000.0
FEM-as input	0.3593	0.1823	21.8	3345.6	72,936.8
-	2.7%	8.8%	6.0%	4.4%	1.3%
DIC, grid 400 × 400	0.3523	0.1935	18.4	3902.6	71,908.1
-	0.7%	3.3%	10.4%	11.5%	0.1%
DIC, grid 200 × 200	0.3509	0.1949	15.8	4493.4	70,801.7
-	0.3%	2.5%	23.4%	28.4%	1.7%
DIC, grid 100 × 100	0.3449	0.2045	13.5	5162.7	69,565.6
-	1.5%	2.3%	34.5%	47.5%	3.4%

**Table 6:** Results of the identification of constituents properties for Material 2.

	υ_m_	υ_f_	R	E_m_, MPa	E_f_, MPa
FEM-reference solution	0.3900	0.2800	44.2	2600.0	115,000.0
FEM-as input	0.3828	0.2897	50.5	2312.1	116,646.7
-	1.9%	3.5%	14.1%	11.1%	1.4%
DIC, grid 400 × 400	0.3748	0.3027	35.1	3270.6	114,869.3
-	3.9%	8.1%	20.6%	25.8%	0.1%
DIC, grid 200 × 200	0.3692	0.3107	25.9	4354.8	112,857.5
-	5.3%	11.0%	41.4%	67.5%	1.9%
DIC, grid 100 × 100	0.3650	0.3175	22.9	4886.8	111,872.9
-	6.4%	13.4%	48.2%	88.0%	2.7%
